# Supplementary material for: A Likelihood Approach for Real-Time Calibration of Stochastic Compartmental Epidemic Models
Source: PLoS Comput Biol. 2017 Jan 17;13(1):e1005257. doi: 10.1371/journal.pcbi.1005257 (PMC5240920; doi:10.1371/journal.pcbi.1005257)
Supplement: S1 File — (TAR.GZ) [file pcbi.1005257.s014.tar.gz › HSPH_Online-SI-Revision/PF08/n1-extreme/n1-extreme_PF_table0.pdf]

1	R0	{2.95019, 14.2854}	{2.07348, 13.5762}	{{1.24719, 7.55154}, {2.8833, 19.1478}}	{29.7955, 24.0688}	inf	302.	124.013	{19.6779, 226.582}	58.9362	0.533944	newly	137.	58.02	{15., 98.}	57.6496	0.503852	Reff	0.882107	0.880099	{0.463842, 1.2832}	30.3699	ar	924.	737.42	{398., 928.}	20.5628
2	R0	{2.67565, 16.946}	{2.10566, 15.6937}	{{1.39638, 11.1314}, {2.74449, 19.7334}}	{23.382, 16.1669}	inf	233.	112.271	{37.6565, 206.864}	52.2273	0.390862	newly	80.	46.74	{19., 72.}	43.3	0.293104	Reff	0.559211	0.89307	{0.552065, 1.21607}	61.8554	ar	926.	912.04	{683., 1048.}	12.2246
3	R0	{2.40066, 16.6543}	{1.8336, 13.1688}	{{1.16966, 6.62607}, {2.56757, 18.8228}}	{26.9663, 26.1863}	inf	237.	86.8048	{12.7464, 148.987}	63.6113	0.606993	newly	91.	42.47	{9., 74.}	53.9451	0.462487	Reff	1.01788	0.910562	{0.483018, 1.44712}	30.1041	ar	916.	668.33	{370., 887.}	27.1605
4	R0	{1.9294, 15.4361}	{1.74714, 13.5997}	{{1.14345, 7.48339}, {2.59907, 19.6025}}	{24.06, 25.3474}	inf	135.	77.0761	{6.38619, 184.451}	57.4241	0.516078	newly	58.	34.22	{5., 72.}	53.3448	0.477253	Reff	1.07853	0.860377	{0.507941, 1.16245}	27.0192	ar	796.	614.61	{248., 887.}	28.0415
5	R0	{2.92859, 19.5393}	{1.67649, 14.6064}	{{1.2203, 7.70014}, {2.3212, 20.}}	{42.7792, 26.0478}	inf	261.	62.1407	{17.6121, 136.331}	76.1913	0.74473	newly	98.	26.08	{11., 46.}	73.3878	0.665003	Reff	0.66479	0.699107	{0.392872, 0.957836}	25.7441	ar	927.	756.94	{491., 981.}	20.9536
6	R0	{1.79622, 12.3148}	{1.71534, 13.426}	{{1.14292, 6.62607}, {2.31231, 19.3211}}	{21.2251, 31.9004}	inf	97.	58.4612	{3.57163, 127.359}	56.3725	0.528375	newly	44.	25.91	{3., 58.}	56.1591	0.47381	Reff	0.941219	0.766171	{0.426152, 1.0551}	28.384	ar	734.	621.6	{274., 866.}	24.6022
7	R0	{2.45855, 17.1776}	{1.84794, 13.312}	{{1.21725, 8.23415}, {2.46976, 18.4377}}	{27.1191, 25.9169}	inf	208.	89.438	{18.4143, 177.453}	57.5036	0.495105	newly	81.	41.35	{14., 64.}	50.5556	0.377912	Reff	0.98342	0.833431	{0.48168, 1.19587}	27.3607	ar	871.	687.15	{375., 892.}	22.1665
8	R0	{2.20009, 15.519}	{1.77995, 15.0141}	{{1.2532, 8.32288}, {2.40871, 20.}}	{25.2212, 22.9692}	inf	171.	79.6051	{17.2722, 163.896}	55.7304	0.467975	newly	72.	31.99	{9., 60.}	56.6528	0.463075	Reff	0.708429	0.763774	{0.440385, 1.12347}	29.5579	ar	839.	802.65	{551., 1026.}	18.5733
9	R0	{2.06493, 9.6414}	{1.90176, 13.3075}	{{1.30044, 8.72462}, {2.55942, 18.5502}}	{22.08, 45.3213}	inf	125.	99.227	{13.7882, 200.204}	48.9505	0.348936	newly	96.	48.69	{8., 91.}	50.4479	0.421036	Reff	1.08409	0.955507	{0.557948, 1.37998}	29.2794	ar	777.	693.74	{428., 899.}	20.9189
10	R0	{2.60775, 19.6936}	{1.69991, 15.01}	{{1.19222, 8.01765}, {2.16374, 20.}}	{35.3873, 24.4825}	inf	215.	81.3586	{23.9915, 162.756}	62.7876	0.548135	newly	62.	33.43	{10., 57.}	48.7903	0.35717	Reff	0.904889	0.765142	{0.471003, 1.01314}	22.375	ar	889.	775.64	{505., 988.}	18.0045
11	R0	{1.99168, 8.13555}	{2.01266, 13.8449}	{{1.3618, 8.51468}, {2.76795, 18.4749}}	{21.4044, 73.025}	inf	102.	100.856	{18.8963, 181.996}	48.197	0.265992	newly	81.	46.8	{13., 86.}	46.321	0.335393	Reff	0.938081	0.833797	{0.403675, 1.2746}	32.2818	ar	772.	736.76	{444., 913.}	17.7539
12	R0	{1.79304, 9.18941}	{1.8197, 12.8556}	{{1.12882, 5.62019}, {2.7346, 17.9172}}	{26.2513, 56.4025}	inf	134.	93.3249	{8.65673, 200.204}	50.6266	0.433618	newly	109.	44.84	{6., 82.}	59.0275	0.534165	Reff	1.0059	0.932543	{0.557948, 1.26061}	25.2209	ar	725.	643.21	{213., 899.}	27.5738
13	R0	{1.84016, 18.3431}	{1.70599, 15.165}	{{1.20529, 8.12987}, {2.37544, 20.}}	{22.5385, 21.1031}	inf	100.	64.4659	{20.3087, 133.913}	53.3883	0.365852	newly	41.	26.33	{11., 52.}	48.2195	0.322513	Reff	1.13354	0.749395	{0.418251, 1.06456}	34.1637	ar	762.	761.1	{510., 965.}	20.601
14	R0	{1.94221, 9.85971}	{2.05518, 14.6134}	{{1.36775, 7.38867}, {2.74643, 20.}}	{22.3147, 56.3992}	inf	139.	114.457	{29.5209, 211.491}	42.7944	0.270972	newly	92.	51.46	{18., 87.}	45.4565	0.320714	Reff	0.594316	0.914756	{0.578675, 1.2634}	56.5321	ar	805.	881.96	{619., 1056.}	20.9043
15	R0	{1.78578, 12.0541}	{1.68037, 15.7764}	{{1.20767, 7.87409}, {2.30428, 20.}}	{21.9328, 44.1096}	inf	118.	73.6157	{20.3023, 142.875}	49.9162	0.362263	newly	65.	28.78	{12., 49.}	57.5692	0.445882	Reff	0.878604	0.760359	{0.439773, 1.03098}	22.8691	ar	732.	754.48	{504., 1000.}	21.5
16	R0	{1.94463, 15.6503}	{1.82952, 13.4691}	{{1.18979, 6.8071}, {2.58092, 18.4377}}	{22.7692, 22.7553}	inf	143.	88.9379	{13.9467, 179.118}	46.2861	0.417388	newly	51.	41.12	{9., 71.}	40.5882	0.299421	Reff	0.958703	0.886761	{0.494194, 1.2746}	26.4153	ar	757.	661.35	{289., 898.}	23.6816
17	R0	{2.08901, 16.4314}	{1.77255, 14.7565}	{{1.22581, 8.17423}, {2.38492, 19.6227}}	{21.6519, 20.5473}	inf	160.	90.4134	{27.5212, 161.636}	49.7623	0.367314	newly	61.	39.77	{15., 71.}	44.3443	0.287351	Reff	1.04868	0.892243	{0.584531, 1.20174}	24.2673	ar	826.	802.29	{522., 1010.}	15.977
18	R0	{2.76419, 18.56}	{1.86784, 13.4555}	{{1.21937, 6.0993}, {2.73737, 19.0531}}	{32.8726, 28.8394}	inf	275.	94.9604	{14.9829, 183.351}	65.478	0.626664	newly	103.	44.62	{10., 72.}	56.9126	0.491749	Reff	0.931532	0.894329	{0.515298, 1.3489}	25.6884	ar	920.	680.01	{336., 903.}	26.2315
19	R0	{2.3821, 17.1399}	{1.80316, 12.9351}	{{1.15409, 6.0993}, {2.46976, 17.9172}}	{27.2768, 27.3476}	inf	229.	93.7508	{14.3638, 182.339}	59.2435	0.550348	newly	77.	46.39	{12., 81.}	44.4026	0.355048	Reff	1.07433	0.928439	{0.558655, 1.26061}	26.2523	ar	883.	658.18	{260., 890.}	26.0747
20	R0	{2.78314, 19.6127}	{1.83522, 14.598}	{{1.32806, 8.17619}, {2.38841, 20.}}	{34.0592, 26.2358}	inf	236.	81.7322	{21.9963, 164.045}	65.3677	0.572402	newly	84.	34.49	{13., 58.}	58.9405	0.453829	Reff	0.411905	0.708661	{0.415143, 0.994832}	74.2578	ar	943.	841.91	{576., 1025.}	15.3097
21	R0	{1.85124, 14.6794}	{1.80051, 14.1404}	{{1.25082, 7.45051}, {2.39176, 19.9848}}	{20.855, 26.0719}	inf	97.	94.6013	{21.9639, 220.541}	57.8683	0.303452	newly	43.	41.76	{15., 80.}	47.7674	0.226553	Reff	1.20516	0.887473	{0.558904, 1.16205}	29.8799	ar	730.	807.81	{522., 1020.}	23.7466
22	R0	{2.1028, 14.9852}	{1.86655, 13.6738}	{{1.31748, 8.23415}, {2.46976, 18.4377}}	{20.0737, 22.0428}	inf	167.	83.2409	{8.16557, 158.073}	54.5267	0.501875	newly	63.	36.95	{5., 72.}	48.8095	0.409222	Reff	0.738083	0.754942	{0.473943, 1.04668}	26.4288	ar	840.	696.3	{408., 885.}	19.5762
23	R0	{1.86601, 10.2157}	{1.81578, 13.2195}	{{1.07874, 6.8071}, {2.46976, 18.6082}}	{23.2772, 43.5062}	inf	118.	85.8216	{7.29603, 179.011}	51.4435	0.446555	newly	79.	40.34	{4., 75.}	51.8481	0.478212	Reff	1.02071	0.935028	{0.550298, 1.45426}	28.762	ar	747.	648.63	{216., 889.}	25.6185
24	R0	{2.34032, 10.0992}	{2.07161, 13.8309}	{{1.24719, 6.62607}, {2.88523, 19.0665}}	{21.1952, 49.1377}	inf	202.	122.578	{11.5712, 218.811}	43.3713	0.392768	newly	137.	55.39	{12., 92.}	59.5693	0.504692	Reff	0.840175	0.832606	{0.463842, 1.23355}	30.3097	ar	887.	742.61	{398., 928.}	18.2694
25	R0	{1.91548, 15.3401}	{1.71153, 13.2625}	{{1.14345, 6.74711}, {2.30907, 19.0443}}	{22.6401, 26.7251}	inf	146.	73.6832	{5.62653, 165.565}	59.0853	0.567218	newly	68.	32.8	{5., 66.}	55.5588	0.511877	Reff	1.10523	0.856156	{0.511557, 1.16245}	27.9251	ar	765.	609.59	{248., 867.}	27.0444
26	R0	{2.75283, 10.6721}	{1.99174, 13.9257}	{{1.32595, 8.39294}, {2.78218, 18.4749}}	{28.5545, 38.4753}	inf	255.	121.65	{28.4581, 209.75}	52.6227	0.414114	newly	153.	56.73	{19., 89.}	62.9216	0.497543	Reff	0.743264	0.818565	{0.517366, 1.0532}	25.2418	ar	926.	739.01	{445., 920.}	20.2819
27	R0	{2.08367, 19.7849}	{1.74429, 14.1993}	{{1.26135, 7.44056}, {2.38742, 20.}}	{22.4453, 28.5129}	inf	158.	77.597	{23.4615, 143.484}	56.7316	0.44306	newly	54.	34.45	{13., 59.}	45.3148	0.302417	Reff	0.927233	0.831007	{0.514639, 1.13725}	23.7926	ar	833.	799.7	{567., 1010.}	16.7227
28	R0	{2.64334, 17.1765}	{1.91366, 13.4977}	{{1.30094, 7.10524}, {2.73737, 18.4749}}	{29.2715, 24.6665}	inf	230.	87.299	{13.5087, 152.99}	62.6471	0.565607	newly	99.	38.95	{8., 63.}	60.6566	0.495362	Reff	0.734849	0.756664	{0.395666, 1.05341}	27.763	ar	934.	706.87	{404., 909.}	24.3201
29	R0	{2.87008, 13.3872}	{1.84973, 14.7204}	{{1.28301, 7.90081}, {2.54472, 20.}}	{35.9095, 27.4276}	inf	267.	107.602	{30.1091, 206.727}	60.1915	0.504962	newly	113.	46.76	{18., 85.}	58.6195	0.466114	Reff	0.608457	0.815036	{0.484232, 1.16878}	41.4664	ar	941.	827.66	{598., 1031.}	16.542
30	R0	{2.06105, 19.6275}	{1.74445, 14.4981}	{{1.23694, 7.77108}, {2.39189, 20.}}	{24.3401, 26.7377}	inf	100.	48.7338	{6.4575, 103.754}	55.8138	0.531859	newly	41.	20.09	{4., 38.}	54.8049	0.470241	Reff	0.715184	0.657095	{0.339427, 0.889513}	24.4373	ar	747.	785.02	{520., 1005.}	21.6493
31	R0	{2.30764, 13.435}	{1.96089, 13.912}	{{1.32613, 9.35432}, {2.8568, 18.7084}}	{23.8609, 21.5833}	inf	231.	101.464	{25.8176, 178.047}	57.0197	0.478514	newly	94.	46.23	{16., 79.}	51.6702	0.411903	Reff	0.717676	0.828562	{0.420704, 1.1636}	37.2746	ar	877.	714.5	{447., 913.}	20.3147
32	R0	{2.30565, 11.9237}	{1.92084, 13.3731}	{{1.28596, 7.55154}, {2.73737, 18.9272}}	{24.0013, 29.9657}	inf	219.	99.5076	{15.7904, 181.996}	55.2564	0.50154	newly	128.	46.46	{9., 79.}	63.7031	0.557848	Reff	0.788532	0.814301	{0.503703, 1.13322}	28.113	ar	853.	707.34	{433., 905.}	19.8195
33	R0	{2.24031, 18.582}	{1.73205, 14.3311}	{{1.24298, 7.89817}, {2.38499, 20.}}	{27.5839, 25.5473}	inf	205.	86.9478	{25.7461, 175.135}	59.7473	0.480435	newly	78.	37.68	{14., 67.}	53.4872	0.38862	Reff	1.10895	0.814609	{0.459878, 1.13615}	28.7276	ar	864.	785.45	{548., 1020.}	18.5637
34	R0	{2.33306, 15.9759}	{1.82338, 13.6829}	{{1.13176, 7.10524}, {2.46976, 18.7084}}	{25.1571, 22.8907}	inf	204.	87.1624	{6.62085, 163.989}	58.1831	0.574238	newly	96.	40.1	{5., 79.}	58.375	0.545383	Reff	0.874898	0.865596	{0.537882, 1.23373}	25.9481	ar	854.	667.79	{260., 873.}	23.1323
35	R0	{2.48357, 18.3573}	{1.77566, 12.7261}	{{1.13176, 6.77341}, {2.46976, 17.5057}}	{29.7211, 31.52}	inf	218.	83.2154	{8.85979, 164.906}	61.8278	0.629642	newly	79.	39.75	{4., 71.}	51.5316	0.480419	Reff	1.0282	0.882253	{0.545374, 1.24956}	26.41	ar	835.	644.2	{248., 885.}	25.2503
36	R0	{2.59548, 19.9046}	{1.86111, 13.7386}	{{1.15964, 6.77341}, {2.75598, 19.2972}}	{30.2893, 30.9913}	inf	240.	84.4913	{4.48368, 179.495}	66.0137	0.751566	newly	83.	37.65	{3., 78.}	60.8554	0.657793	Reff	0.931777	0.805587	{0.428419, 1.13664}	26.1746	ar	863.	666.68	{274., 917.}	25.1889
37	R0	{2.29641, 16.9868}	{1.699, 15.0486}	{{1.22112, 8.15084}, {2.29374, 20.}}	{28.2334, 20.9302}	inf	186.	67.7835	{18.0202, 134.64}	63.6044	0.558148	newly	65.	27.75	{10., 51.}	57.4615	0.449435	Reff	0.803743	0.708332	{0.408567, 0.96318}	22.5496	ar	877.	774.94	{533., 972.}	17.48
38	R0	{2.65743, 15.4569}	{2.00895, 15.6081}	{{1.30915, 9.76241}, {2.64179, 20.}}	{25.9079, 21.6795}	inf	233.	102.439	{28.8141, 205.278}	57.4489	0.453615	newly	114.	44.94	{16., 81.}	61.5439	0.481752	Reff	0.733451	0.94939	{0.568995, 1.29935}	39.7198	ar	897.	882.41	{639., 1046.}	14.4114
39	R0	{2.67953, 14.5132}	{1.83469, 13.7889}	{{1.07874, 6.62607}, {2.46976, 19.1478}}	{32.1567, 24.9218}	inf	233.	97.755	{4.61248, 187.654}	58.5729	0.610142	newly	117.	44.7	{4., 81.}	61.7949	0.606148	Reff	1.15488	0.951336	{0.581194, 1.40199}	27.6842	ar	894.	666.58	{238., 885.}	25.8143
40	R0	{1.94594, 11.928}	{1.8821, 14.0928}	{{1.21725, 8.42022}, {2.5458, 18.3794}}	{21.4157, 31.5861}	inf	115.	80.7876	{13.6259, 158.073}	48.2367	0.373315	newly	66.	35.44	{8., 69.}	50.6061	0.409098	Reff	0.8387	0.753576	{0.387145, 1.07361}	28.948	ar	796.	684.69	{327., 891.}	21.1621
41	R0	{1.66297, 10.0047}	{1.67204, 14.526}	{{1.19876, 7.48836}, {2.3631, 20.}}	{20.5427, 57.132}	inf	73.	59.4217	{9.85214, 120.404}	49.5437	0.349923	newly	55.	23.96	{8., 42.}	56.9818	0.458723	Reff	0.964523	0.750428	{0.431408, 1.05258}	26.6843	ar	716.	758.85	{517., 1001.}	21.574
42	R0	{2.91568, 16.4453}	{2.05055, 13.5066}	{{1.31748, 7.55154}, {2.86193, 19.0269}}	{29.7857, 23.8109}	inf	275.	118.855	{22.7047, 210.945}	56.8235	0.487698	newly	105.	55.33	{16., 89.}	47.7238	0.358857	Reff	0.906776	0.851846	{0.460861, 1.26061}	28.625	ar	919.	736.02	{421., 923.}	20.2894
43	R0	{2.55898, 15.8889}	{1.94476, 14.0749}	{{1.33113, 8.42022}, {2.73737, 18.7523}}	{26.8491, 20.8004}	inf	219.	100.139	{18.4488, 179.118}	54.5691	0.463253	newly	75.	45.08	{12., 71.}	43.28	0.326997	Reff	1.07989	0.874761	{0.484814, 1.33161}	30.7983	ar	904.	712.98	{384., 905.}	21.6217
44	R0	{2.28649, 15.9017}	{1.94312, 14.2999}	{{1.30044, 8.71343}, {2.47514, 19.1171}}	{20.6165, 20.7943}	inf	219.	94.621	{12.0788, 172.84}	57.4419	0.502565	newly	76.	40.72	{10., 71.}	48.6842	0.382094	Reff	0.756828	0.720079	{0.420779, 1.03189}	26.3855	ar	880.	728.33	{468., 891.}	18.058
45	R0	{2.65121, 18.6339}	{1.95823, 13.442}	{{1.31242, 8.33653}, {2.8568, 18.4249}}	{28.6341, 28.4271}	inf	260.	105.658	{20.4579, 202.043}	59.6447	0.541001	newly	105.	49.88	{11., 91.}	53.1429	0.44867	Reff	1.01011	0.903695	{0.494077, 1.29249}	29.0307	ar	928.	709.5	{459., 918.}	23.7349
46	R0	{2.52291, 18.0251}	{1.81952, 13.8798}	{{1.15921, 8.09734}, {2.43839, 19.4054}}	{29.2818, 26.0089}	inf	228.	91.3927	{16.5786, 184.486}	59.9941	0.534924	newly	79.	40.9	{11., 71.}	51.1646	0.389032	Reff	0.915816	0.826763	{0.519068, 1.11676}	24.304	ar	894.	681.03	{374., 884.}	24.2718
47	R0	{2.22618, 17.1225}	{1.81809, 13.3088}	{{1.18979, 5.72868}, {2.59755, 19.1171}}	{24.8826, 26.9935}	inf	176.	82.8718	{8.84123, 154.78}	54.9304	0.524509	newly	67.	39.32	{9., 73.}	48.209	0.402709	Reff	1.15316	0.890829	{0.500072, 1.33593}	32.9153	ar	850.	658.91	{332., 902.}	24.7965
48	R0	{2.04405, 16.073}	{1.76473, 13.3035}	{{1.14345, 6.0993}, {2.59755, 19.1171}}	{25.5221, 25.7319}	inf	137.	73.1111	{5.2926, 146.171}	51.7954	0.527419	newly	68.	34.56	{3., 71.}	54.5588	0.541987	Reff	1.11196	0.888023	{0.429818, 1.33593}	31.9324	ar	779.	629.46	{276., 896.}	26.4262
49	R0	{2.84396, 18.097}	{2.03616, 13.5585}	{{1.18979, 8.02695}, {2.81617, 18.3794}}	{28.9024, 26.2575}	inf	289.	116.154	{21.7801, 208.199}	59.8083	0.514735	newly	115.	54.26	{15., 91.}	52.8174	0.411334	Reff	0.665487	0.797213	{0.436659, 1.14701}	35.0408	ar	952.	735.48	{363., 918.}	22.7437
50	R0	{1.85341, 18.0975}	{1.79264, 13.7813}	{{1.07874, 8.23415}, {2.45983, 18.8228}}	{21.5364, 25.7991}	inf	148.	74.8925	{4.33027, 148.987}	53.6669	0.545559	newly	54.	33.75	{3., 70.}	50.2037	0.454327	Reff	0.850715	0.840632	{0.441522, 1.23515}	30.9817	ar	754.	647.86	{242., 881.}	23.5782
